# Immigrant Medicine

**DOI:** 10.3201/eid1406.080154

**Published:** 2008-06

**Authors:** Patricia F. Walker, Elizabeth D. Barnett, Fern R. Hauck, Richard D. Pearson

**Affiliations:** * University of Virginia School of Medicine, Charlottesville, Virginia, USA

The United States is experiencing its largest wave of immigration since the beginning of the 20th century, and immigrants are making their way to every region, city, and town. As a result, medical professionals are increasingly called on to care for persons of diverse cultures and ethnicity. Providing care that is culturally sensitive and appropriate is a challenge. In addition to substantial language, cultural, legal, and financial barriers, physicians are faced with medical conditions that they may not have seen before, including unusual infectious diseases and complex, heartbreaking mental health and social issues.

Immigrant Medicine provides, in 1 handy, compact reference, a comprehensive discussion of the issues involved in the compassionate and competent care of refugees and immigrants. The 78 contributors to this reference represent a “Who’s Who” of experts in their respective fields. The book is divided into 8 sections on immigrant medicine: 1) introduction; 2) medical screenings and immunizations; 3) epidemiology of diseases and disorders; 4) major diseases and disorders; 5) additional diseases and disorders; 6) chronic illness; 7) mental health and illness; and 8) special issues.

The editors begin with an excellent introduction on the magnitude and scope of immigrant health. The second chapter, “Compassion,” is unique in that the author, David R. Shim, proposes that compassion can be learned. He challenges the reader to contemplate the suffering that so many in the world are forced to endure and the difference between acting more compassionately and genuinely being more compassionate. For the most part, subsequent chapters and content flow in a logical sequence to such key areas as cultural competence before moving on to the diagnosis, treatment, and prevention of specific diseases and disorders.

Topics are relevant to practitioners new to immigrant medicine as well as those who are more experienced. For the clinician outside of academic medical centers or special immigrant clinics, language services and payment often present substantial barriers to care. The chapters on language assistance and communicating with patients who have a limited understanding of English describe a more “nuts and bolts” approach to accessing and financing interpreter services, in addition to the legal requirements for language assistance.

Sections 3–6 are particularly helpful for physicians who treat immigrants. Provided in these sections are expanded differential diagnoses by organ system, country of origin, latency period, and race and ethnicity for syndromes such as fever, diarrhea, eosinophilia, and skin problems. Also included are diseases not normally considered in residents of North America. Specific infectious diseases are described in detail, although some diseases are intermingled with syndromes in one section, and additional diseases and disorders are listed alphabetically in another. Chapters on individual diseases are well written. Summaries of therapeutic options are provided, but understandably do not address all of the nuances of treatment. No one textbook can stay abreast of recent advances and changing recommendations. Physicians lacking experience in the diagnosis and treatment of these diseases may need to access additional therapeutic information in the literature or request the recommendations of infectious diseases experts.

A notable strength of this book is the inclusion of the full range of other conditions likely to be seen by those caring for immigrants and refugees. Included are discussions on women’s health, dental diseases, and preventive healthcare, which are often overlooked. Because psychiatric and other mental health resources are limited in many locations, the chapters on mental illness and on survivors of torture and violence toward women are of considerable value. Finally, Section 8 provides information on topics frequently missing from other publications, such as school readiness, health literacy, and healthcare risks that immigrants face when they visit friends and relatives abroad. The last chapter includes an excellent table that lists pretravel health resources (websites). It would have been useful to have more of this type of web-based resource information highlighted in other chapters. Overall, we highly recommend Immigrant Medicine for clinicians, other healthcare providers, and public health departments who care for immigrants and refugees.

